# Tillage and irrigation increase wheat root systems at deep soil layer and grain yields in lime concretion black soil

**DOI:** 10.1038/s41598-021-85588-6

**Published:** 2021-03-18

**Authors:** Jinfeng Wang, Zhuangzhuang Wang, Fengxu Gu, Huan Liu, Guozhang Kang, Wei Feng, Yonghua Wang, Tiancai Guo

**Affiliations:** 1 Agronomy College of Henan Agricultural University, #15 Longzihu College District, Zhengzhou, 450046 China; 2 National Engineering Research Centre for Wheat, #15 Longzihu College District, Zhengzhou, 450046 China; 3 Collaborative Innovation Centre of Henan Grain Crops, #15 Longzihu College District, Zhengzhou, 450046 China

**Keywords:** Plant development, Plant physiology

## Abstract

In lime concretion black soil, a two-factor (tillage and irrigation) split block experiment from 2015 to 2017 was conducted to identify whether their combination is suitable for the improvement of winter wheat yield and water use efficiency. The main treatments were subsoiling (SS) and rotary tillage (RT), with secondary treatments of three irrigation regimes: no irrigation during the whole growth period (W0), irrigation at jointing stage (W1), and irrigation at both jointing and anthesis stages (W2). In combination with a soil column experiment, the contribution of the root system in different soil layers to yield was clarified. The results indicated that both tillage and irrigation significantly influenced the spatiotemporal distributions of the root systems and yield components, while tillage produced the strongest effect. Compared with RT, SS significantly promoted the root penetration and delayed root senescence in deep soil layers. With increasing soil depth, each root configuration parameter (dry root weight density, DRWD; root length density, RLD; root surface area per unit area, RSA; root volume per unit area, RV) gradually decreased, and the peak appearance times of each root parameter in RT and three parameters (RLD, RSA and RV) in SS were postponed from heading to anthesis and from anthesis to filling stage, respectively. The average post-peak attenuation values at soil layers from 60 to 100 cm in W1 were less than those in W0 and W2. SSW1 generated the highest grain yields, with an average increase of 31.88% compared with the yield in RTW0. Root systems at three soil layers (0–40 cm, 40–80 cm and below 80 cm) differentially contributed to grain yields with 78.32%, 12.09% and 9.59%, respectively. The growth peak of the deep root system in SSW1 was postponed to the filling stage, and the post-peak attenuation declining rates were also slowed. Therefore, SSW1 is an effective cultivation method improving grain yields and water use efficiency in lime concretion black soil.

## Introduction

Wheat (*Triticum aestivum* L.), one of the most important food crops in the world, is the main source of daily protein and provides 20% calories consumed by humans^1^. China has the largest wheat growth areas and productions with 2.3 × 10^7^ ha and 1.3 × 10^8^ t in 2020, respectively. There are about 3.2 × 10^6^ ha lime concretion black soil in China, and it accounts for 13.9% of wheat growth areas and is mainly distributed in Anhui, Henan, Shandong and Jiangsu provinces. In this soil type, wheat grain yields are lower over 15% than those in other types of soil, because its groundwater is shallow, and water shortages often occur under dry weather conditions, especially in growth seasons with strong evaporation and high water consumption^2^. During these water shortage periods, the rise of capillary water hardly keeps up with the loss due to evaporation and transpiration in the upper layer of the soil. Thus, the supplementary irrigation is necessary to ensure a high and stable yield. In recent years, successive RT, heavy machinery rolling, and unsuitable land preparation operations have caused changes in the soil structure of most wheat fields, especially those with lime concretion black soil^3,4^. With such operations, the plough layer becomes shallower, the plough pan is thickened and moved upward, soil compaction and bulk density are increased, and the transduction conditions of water, vapour and heat are destroyed^5^. Moreover, soil compaction stress and water conduction obstruction have become important issues affecting wheat root growth, yield formation and the improvement of water and fertilizer utilization efficiency. In addition, the conditions of field irrigation facilities in this region are relatively bad, and most of farmers neglect irrigation. In lime concretion black soil, therefore, agronomic measures such as tillage and irrigation can effectively improve the physical properties, reduce the imbalance between the soil water storage and supply, and create a soil environment conducive to wheat root growth and high efficient water utilization to achieve simultaneous improvement in wheat grain yield and water use efficiency. These are key measurements to improve the comprehensive production performance of winter wheat in this region.

In lime concretion black soil, the composition, structure and porosity of lime concretion black soil are very different from other soils, which cause this soil to have a low water-holding capacity, weak hydraulic conductivity, slow capillary water rising speed and a small rising height^2^. Lime concretion black soil is characterized with undesirable properties, such as high viscosity, dry shrinkage, wet expansion, poor soil structure and short suitable tillage time, which have become the main factors limiting further increases in winter wheat yield^6^.

Previous studies have shown that compared with traditional cultivation, optimized cultivation improves the wheat root distribution, delays wheat root senescence, and promotes the absorption of water and nitrogen in deep soil, all of which increase wheat production^7^. Strip RT after SS is a high-yield, efficient and water-saving tillage practice that can increase farmland water consumption, enhance stored soil water consumption, promote dry matter accumulation and increase the photosynthetic rate and water use efficiency of flag leaves^8–10^. In addition, once or twice suitable irrigations are beneficial to obtain higher yield and water use efficiency^11^, and the irrigation water use efficiency is highest when irrigation is operated at the critical period of water^12^. The CERES-Wheat model can simulate and predict the effects of different irrigation modes. Studies have shown that the application of 75 mm of irrigation water during jointing and anthesis is the best irrigation strategy in the North China Plain, and it is believed that one irrigation event at the jointing stage can be used as an alternative irrigation mode to reduce irrigation and maintain the sustainable development of farmland water resources^13^. In semi-arid areas, the ridge-furrow rainfall harvesting system combined with 75 mm of irrigation increases soil moisture across the rooting area, making it a high-yield and efficient water-saving strategy^14^.

In previous studies, the effects of single factor, such as variety^15^, planting density^16–18^, cultivation pattern^19^, fertilizer application^20,21^, irrigation amount, and irrigation frequency^22^, on root growth and wheat development were examined. The researches about irrigation-tillage mostly focused on soil properties^23^, greenhouse gas emissions^24^, water use efficiency and yield^6^. Gajri and his colleagues showed that deep tillage and early irrigation could short the time needed for root to reach a specified depth, thus improving water use efficiency and achieving high yield^25^. For lime concretion black soil, it has been shown that excessive irrigation is not conducive to the efficient use of water and the combination of SS and one irrigation event at jointing is a suitable cultivation pattern that results in simultaneous increases in winter wheat yield and water use efficiency in this region^6^. However, the regulatory effects of tillage and irrigation on the root parameters of winter wheat remain unclear in different soil layers in lime concretion black soil. In this study, tillage, irrigation, and their interaction were implemented in the field conditions of the lime concretion black soil for two wheat growth seasons. To evaluate the contribution of roots in different soil layers to grain yields, in addition, soil column experiment was also conducted. The objective of this study was to explore the effects of tillage and irrigation on root characteristics and grain yields in lime concretion black soil, and clarify their regulatory mechanisms underlying the suitable wheat root construction in this soil type.

## Results

### Effects of tillage and irrigation on yield

As shown in Table 1, the significant effects of tillage and irrigation on grain yield and its components during the wheat growing season were similar between the two years. Grain yield and its components in SS were higher than those in the RT treatment, except the kernel number per spike in 2016–2017. However, in both tillage treatments, the yield in W1 was the highest, and that in W0 was the lowest. The average yield in W1 was 22.18% and 24.16% higher than that in W0 under SS and RT, respectively. Moreover, the yield in SSW1 was the highest in the two years, with an average increase of 31.88% compared with the lowest value, which was observed in treatment RTW0. Under a given tillage method, with an increase in the number of irrigation events, spike number showed an increasing trend, thousand-grain weight showed a decreasing trend, and the regularity of kernel number per spike varied. However, while there was no significant difference between W0 and W1 in the first year of the experiment, the kernel number per spike under both the RT and SS conditions reached a maximum value in the W1 treatment in the two years. In 2016–2017, the average kernel number per spike in W1 increased by 21.08% and 8.63%, respectively, compared with that in W0 and W2.

**Table 1:** Effects of tillage practice and irrigation regime on the yield and yield components of winter wheat in lime concretion black soil.

Years	Treatments		Spike number	Kernels per spike	Thousand-grain weight	Yield
			(10^4^·ha^−1^)	(kernel·spike^−1^)	(g)	(kg·ha^−1^)
2015–2016	SS	W0	536.18ab	38.92ab	46.35a	6984.60d
		W1	569.49a	40.48a	43.56bc	8507.85a
		W2	573.53a	37.59bc	42.07 cd	7833.30b
	RT	W0	497.40b	38.70ab	45.41ab	6528.60e
		W1	536.77ab	37.45bc	41.69 cd	8046.90b
		W2	534.39ab	35.60c	40.51d	7536.15c
	Tillage		5.52*	8.93*	8.49*	31.29***
	Irrigation		2.44	6.88*	29.83**	149.69***
	Tillage*Irrigation		0.02	1.97	0.30	0.55
2016–2017	SS	W0	683.98bc	29.43c	44.94a	8129.27c
		W1	732.83ab	36.67a	41.20bc	9958.63a
		W2	770.82a	33.50b	40.01 cd	9277.32b
	RT	W0	659.88c	30.87c	42.74ab	7474.23d
		W1	717.78abc	36.34a	39.97 cd	9339.25b
		W2	726.12ab	33.71b	38.22d	8407.08c
	Tillage		3.22	0.43	6.59*	56.40***
	Irrigation		8.46**	29.97***	16.92**	126.19***
	Tillage*Irrigation		0.32	0.61	0.18	0.68

Data followed by different lowercase letters within any column indicate that the difference is significant at the P = 0.05 level. *, **, and *** indicate significant differences at the P = 0.05, P = 0.01 and P = 0.001 levels, respectively. The same as below.

According to the results of variance analysis, the effects of different combination treatments on thousand-grain weight and grain yield in the two-year test were obvious, while the effects on spike number and kernel number per spike were unstable. The tillage and irrigation obviously affected grain yields mainly by regulating the thousand-grain weight, and the spike number and thousand-grain weight, respectively. However, the interaction effects of tillage and irrigation were insignificant.

### Effects of tillage and irrigation on root development indices and root architecture

The effect of tillage or irrigation on root parameters was significant and in different degree; however, the interaction effect of tillage and irrigation was not significant, and the main regulatory effect was of tillage (Tables 2 and 3). The total dry root weight (TDRW), total root length (TRL), total root surface area (TRSA) and total root volume (TRV) in the SS treatment in each growth period were all higher than those in the RT treatment. Compared with RT, TDRW, TRL, TRSA and TRV parameters in SS increased by 3.52–47.56%, 7.16–46.38%, 6.48–53.14% and 6.22–123.80% (2015–2016), and 6.54–34.38%, 5.16–54.87%, 12.54–49.09%, and 8.52–63.88% (2016–2017), respectively. Moreover, during the two-year test, the TDRW, TRL, TRSA and TRV in W1 were better than those in the other irrigation treatments. In comparison to W0, the above parameters in W1 increased by 1.06–53.78%, 3.33–29.42%, 4.78–60.44%, and 3.27–75.52% (2015–2016), respectively. And similar results appeared in 2016–2017 wheat growth season (Tables 2 and 3). Compared with W2, similar increasing proportions of the above parameters in W1 appeared with 1.71–12.02% and 1.20–8.22%, 1.12–11.02% and 1.33–15.15%, 2.01–11.33% and 0.92–10.57%, and 1.38–9.90% and 1.79–14.01% in two wheat growth seasons (2015–2016 and 2016–2017), respectively.

**Table 2:** Effects of tillage practice and irrigation regime on the total dry root weight and total root length of winter wheat.

Year	Treatment	Total dry root weight (TDRW)/g·m^−2^					Total root length (TRL)/cm·cm^−2^				
		Jointing	Heading	Anthesis	Filling	Maturity	Jointing	Heading	Anthesis	Filling	Maturity
2015–2016	SSW0	49.55a	94.76c	118.30d	83.30c	60.90d	80.54a	129.50d	152.70c	125.69c	93.75b
	SSW1	49.30a	133.77a	171.12a	123.40a	93.17a	83.52a	148.51a	192.73a	167.20a	119.17a
	SSW2	48.50a	130.11ab	162.31b	119.26a	86.87b	83.34a	147.00a	170.45b	157.38b	114.69a
	RTW0	41.74b	85.19d	79.67f.	65.54d	49.19e	72.82b	121.32e	118.00f.	89.83e	77.06c
	RTW1	42.96b	133.85a	124.71c	92.52b	76.12c	74.95b	140.12b	141.08d	111.73d	95.20b
	RTW2	42.21b	127.43b	101.77e	87.12c	71.31c	73.37b	135.16c	130.22e	106.04d	93.37b
	Tillage practice	58.12***	9.97**	1089.74***	582.06***	126.20***	71.45***	66.13***	1124.18***	499.42***	190.14***
	Irrigation regime	0.26	463.73***	384.41***	352.43***	190.89***	2.10	100.46***	209.70***	80.41***	82.31***
	T*I	0.31	4.98*	19.03***	16.96***	1.47	0.40	1.04	15.72***	7.87**	2.02
2016–2017	SSW0	75.81b	110.50e	123.86c	73.27e	64.83d	124.35a	159.49d	204.26a	154.15c	104.28c
	SSW1	78.52a	132.49a	148.36a	109.32a	126.20a	123.82a	188.03a	208.17a	187.22a	143.04a
	SSW2	78.14a	125.77b	144.70b	100.76b	112.78b	124.14a	169.44bc	202.50a	172.13b	121.98b
	RTW0	70.05d	100.55f.	92.49f.	55.26f.	55.80e	114.24d	153.22e	112.63d	94.36f.	73.91e
	RTW1	74.80bc	119.57c	112.24d	93.17c	99.00c	120.55b	172.62b	154.95b	128.75d	102.83c
	RTW2	73.35c	113.88d	105.53e	86.47d	95.33c	117.03c	165.76c	140.70c	108.46e	91.53d
	Tillage practice	65.63***	344.14***	1364.07***	266.60***	147.53***	194.28***	25.99***	1776.30***	943.96***	1397.55***
	Irrigation regime	14.62***	377.81***	192.62***	519.61***	483.49***	11.64***	69.80***	67.13***	97.41***	470.53***
	T*I	1.00	1.95	5.58*	1.18	12.71**	16.38***	4.61*	50.72***	0.62	13.15***

**Table 3:** Effects of tillage practice and irrigation regime on the total root surface area and total root volume of winter wheat.

Year	Treatment	Total root surface area (TRSA)/ × 10^3^ m^2^·ha^−1^					Total root volume (TRV)/m^3^·ha^−1^				
		Jointing	Heading	Anthesis	Filling	Maturity	Jointing	Heading	Anthesis	Filling	Maturity
2015–2016	SSW0	196.49abc	490.33d	576.39c	410.18c	275.59d	78.47b	142.74e	216.82c	151.82c	92.08c
	SSW1	204.29a	583.34a	681.95a	634.77a	489.39a	81.67a	185.52a	254.10a	223.84a	172.63a
	SSW2	201.47ab	556.47b	644.03b	592.47b	440.85b	80.55a	179.14b	238.88b	209.12b	161.56b
	RTW0	183.30d	419.16e	406.78f.	288.32e	220.46e	73.46c	138.20f.	132.35f.	69.76e	49.62f.
	RTW1	193.66bc	551.30b	512.47d	398.64c	306.48c	75.22c	172.28c	173.89d	101.01d	76.08d
	RTW2	188.64 cd	526.67c	494.11e	382.27d	274.00d	74.21c	167.19d	163.43e	97.74d	64.77e
	Tillage practice	30.31***	104.58***	1193.25***	2729.11***	1347.90***	172.87***	79.02***	1096.29***	7385.13***	9831.03***
	Irrigation regime	5.62*	246.76***	179.16***	811.16***	593.28***	10.16**	460.97***	92.84***	674.00***	1689.73***
	T*I	0.13	9.60**	1.92	91.01***	119.48***	1.06	5.91*	1.30	97.94***	519.53***
2016–2017	SSW0	319.66a	596.39c	675.64b	508.28c	348.47c	101.30b	176.42d	244.24c	141.15d	92.54e
	SSW1	323.78a	704.16a	756.40a	666.75a	508.11a	104.98a	216.19a	338.49a	235.63a	180.10a
	SSW2	321.55a	679.16b	759.65a	608.70b	442.01b	103.36ab	204.76b	314.47b	210.74b	160.96b
	RTW0	279.49c	490.64d	414.78e	312.74f.	276.74f.	93.80d	165.59e	166.80f.	115.74e	85.71f.
	RTW1	290.68b	615.46c	558.45c	464.33d	329.74d	96.73c	193.25c	200.68d	154.85c	129.43c
	RTW2	287.32b	599.97c	516.77d	419.30e	315.76e	94.80 cd	176.83d	180.00e	138.28d	110.54d
	Tillage practice	629.27***	209.32***	1044.13***	1196.11***	1523.31***	139.38***	88.41***	4176.27***	1272.93***	2343.77***
	Irrigation regime	9.81**	129.48***	91.66***	259.33***	372.10***	7.73**	79.99***	433.25***	559.35***	2754.79***
	T*I	2.36	1.52	6.68*	0.44	91.74***	0.21	5.40*	117.95***	106.63***	384.49***

During the whole growth period, the root parameters under different combination treatments showed an inverted "V" trend, first increasing and then decreasing in both years. All root parameters in the SS treatment reached a peak at the anthesis stage, while those in the RT treatment mostly reached a peak earlier, at the heading stage. Moreover, four root configuration parameters (TDRW, TRL, TRSA and TRV) were the highest and lowest in SSW1 and RTW0 treatments, respectively.

**Figure 1:**
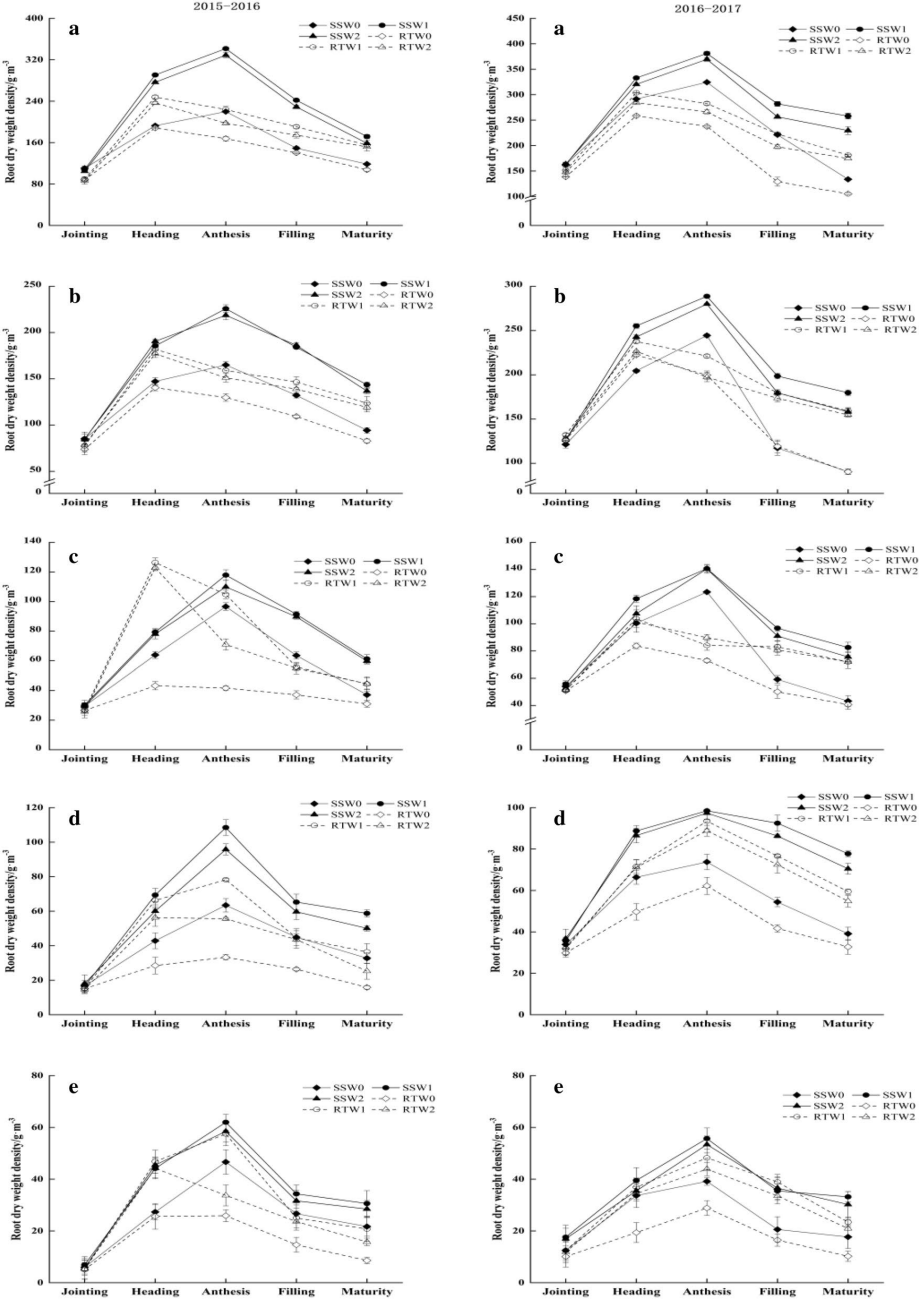
Effects of tillage practice and irrigation regime on the dry root weight density of winter wheat in different soil layers. **Note**: a, b, c, d and e represent the soil layer of 0–20 cm, 20–40 cm, 40–60 cm, 60–80 cm and 80–100 cm, respectively.

**Figure 2:**
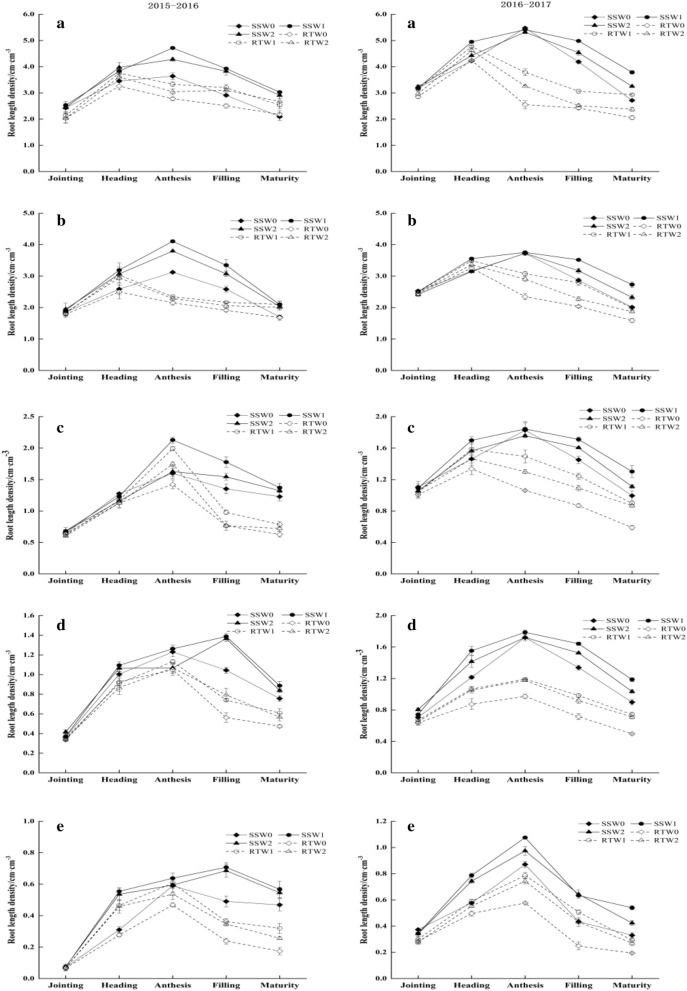
Effects of tillage practice and irrigation regime on the root length density of winter wheat in different soil layers.

**Figure 3:**
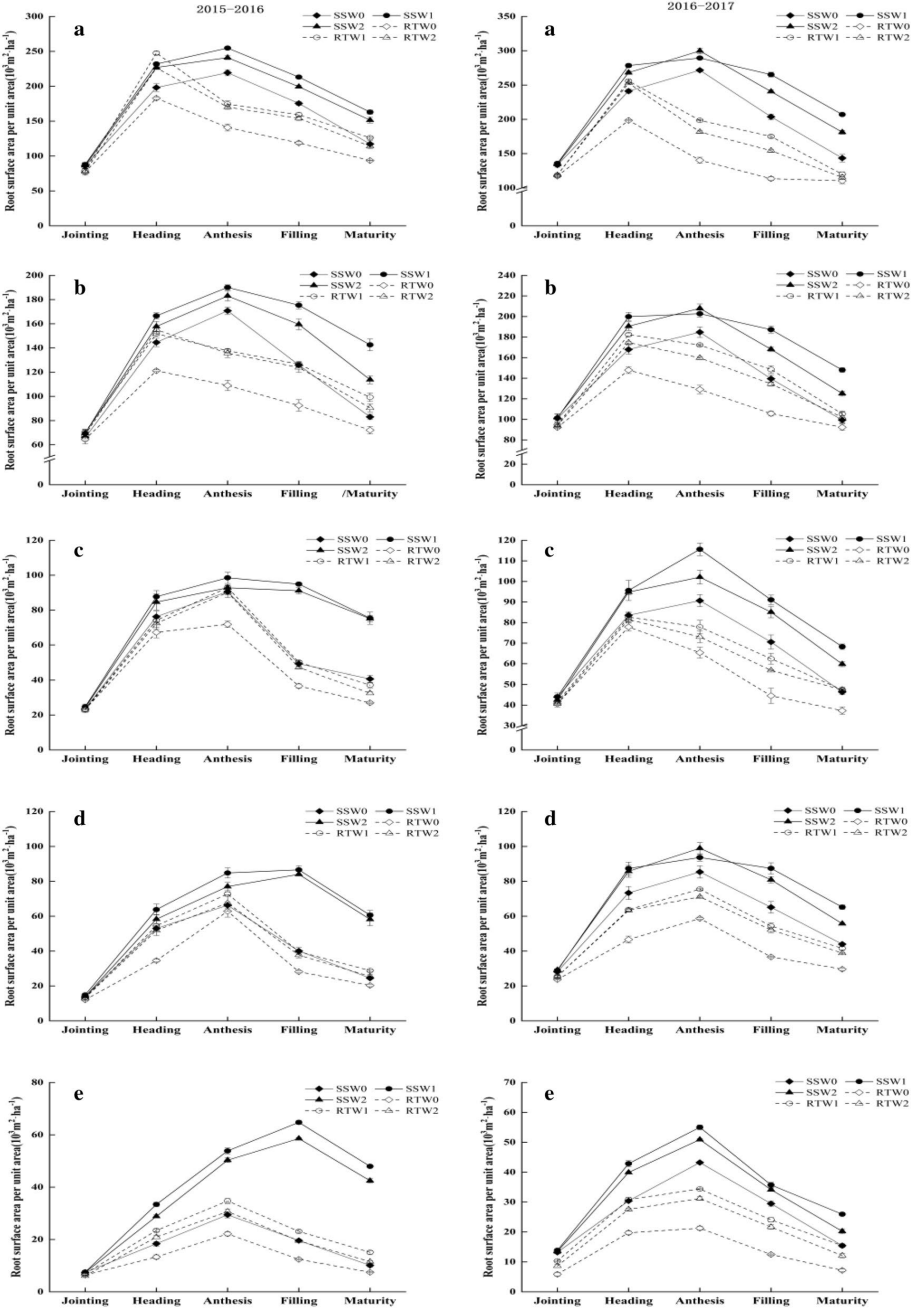
Effects of tillage practice and irrigation regime on the root surface area per unit area of winter wheat in different soil layers.

The figures (Figs. 1; 2; 3; and 4) show that the distributions of root growth characteristic parameters in different soil layers were different, and in the same growth period, the DRWD, RLD, RSA, and RV of different combination treatments gradually decreased as the soil depth increased. Considering the whole growth season, the DRWD, RLD, RSA, and RV in different soil layers for each combination treatment exhibited a single peak curve, rising first and then falling.

**Figure 4:**
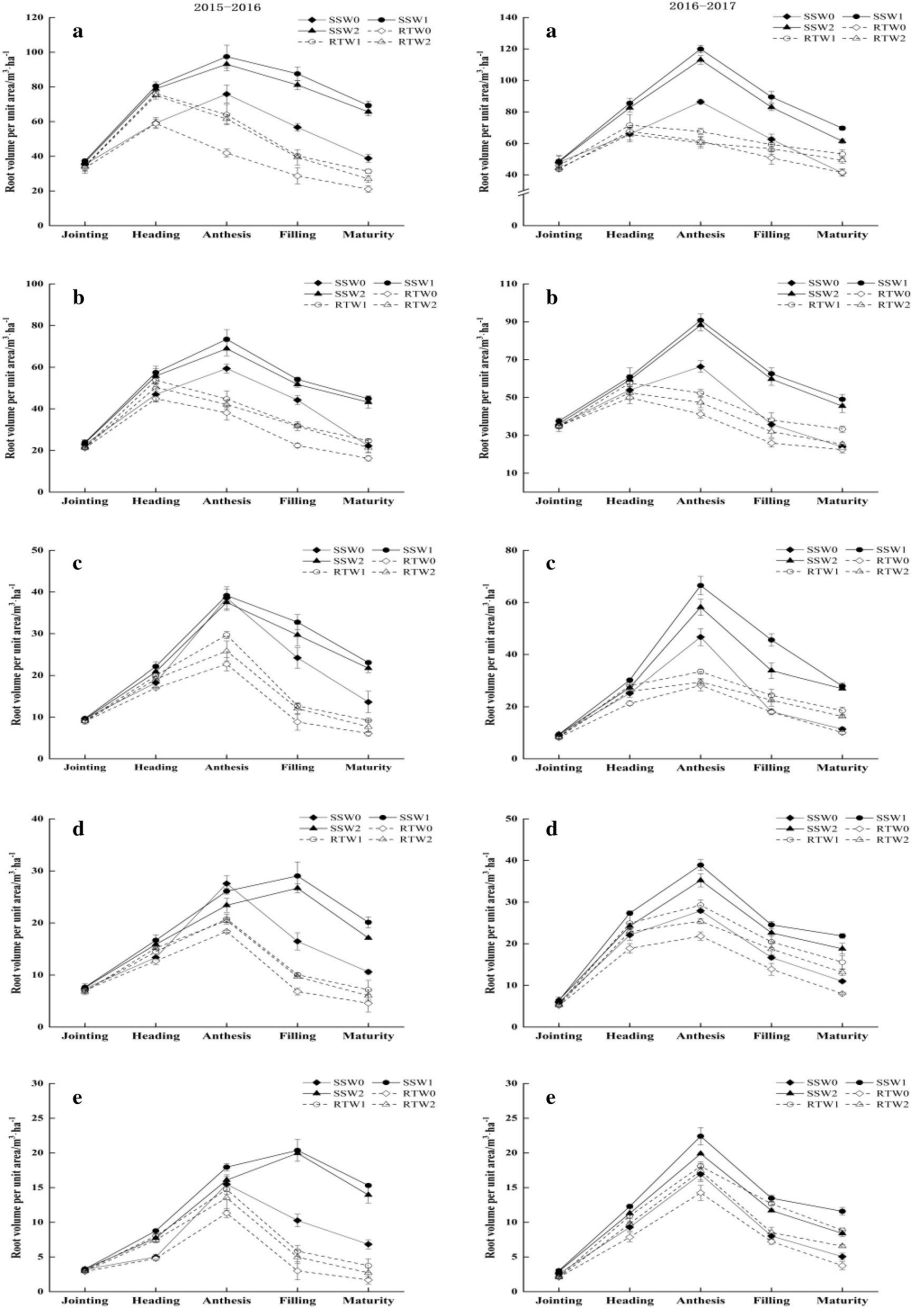
Effects of tillage practice and irrigation regime on the root volume per unit area of winter wheat in different soil layers.

As soil depth increasing, the root parameters varied. In the upper soil layers (0–40 cm), the DRWD, RLD, RSA and RV under the RT treatment all reached their maximum value at the heading stage, while the maximum values under SS were postponed to the anthesis stage. In the middle soil layers (40–60 cm), the maximum values of DRWD and RV under RT appeared at the heading stage and anthesis stage, respectively. The maximum values of RLD and RSA under RT were different in two years, which both appeared at the anthesis stage in 2015–2016 and the heading stage in 2016–2017. However, the peak appearance time of each root parameter under the SS treatment appeared at the anthesis stage. The change in root parameters below the 60 cm soil layers varied depending on the tillage method and growth year. In 2015–2016, the root growth parameters under RT peaked at the anthesis stage, except that of DRWD treated with RTW2, which appeared at heading. The DRWD under SS reached its maximum value at the anthesis stage, and the maximum values of RLD, RSA and RV mostly appeared at the filling stage. However, in 2016–2017, the root parameter peaks in each combination treatment appeared at the anthesis stage. As soil depth increased and the growth period advanced, the peak time of root configuration growth parameters treated with RT tended to be delayed, that is, from the heading stage to the anthesis stage. However, the peak times of RLD, RSA and RV under SS were further delayed to the filling stage.

The effects of tillage and irrigation on root parameters in the middle and upper soil layers were not obvious, but the regulation of roots in the soil below 60 cm was significant. In the deep soil below 60 cm, the post-peak attenuation values of all root parameters in W1 decreased by 22.57% and 7.85% in comparison with W0 and W2, respectively. Compared with RT, the attenuation values under SS were reduced by 23.79% on average. Moreover, the post-peak attenuation values of DRWD, RLD, RSA, and RV in SSW1 were significantly lower than those in other treatments, and especially, they were lower by 31.65%, 40.17%, 44.23% and 50.35% than RTW0, respectively, in which the attenuation decreasing rates were highest.

### Effects of root cutting in different soil layer on yield

Root cutting in different soil layers (no root cutting, CK; at 40 cm below the surface, T-40; at 80 cm below the surface, T-80) had a significant regulatory effect on winter wheat yield, with the T-40 treatment having the strongest regulatory effect (Table 4). The contribution rates of roots in the 0–40 cm, 40–80 cm and below 80 cm soil layers to yield were 78.32%, 12.09% and 9.59%, respectively. Root cutting in different soil layers reduced yield, and compared with that in CK, the grain yield in T-40 and T-80 decreased by 21.68% and 9.59%, respectively. Between T-40 and T-80, the significant differences in kernel number per spike and grain weight were caused by 40–80 cm root system, and between CK and T-80, the significant difference in grain weight was mainly due to the root below 80 cm (Table 4). Thus, the root system in the 40–80 cm soil layer regulated the kernel number per spike and grain weight of wheat, while the roots below 80 cm mainly regulated grain weight to affect the yield.

**Table 4:** Effects of cutting roots in different soil layers on the yield and yield components of winter wheat.

Treatment	Spike number (number/column)	Kernel per spike (kernel/spike)	Thousand-grain weight (g)	Yield (g/column)
CK	46.41a	38.08a	44.11a	76.10a
T-40	45.73a	33.44b	37.67c	59.64c
T-80	45.43a	37.81a	40.46b	68.76b

### The relationships among root parameters, grain yield, soil water content, soil water use efficiency and precipitation water use efficiency

According to the correlation analyses between root growth parameters and grain yield (see Supplementary Fig. S1 online), the growth parameters TDRW, TRL, TRSA and TRV were positively correlated with grain yield to different degrees during each growth period of winter wheat. For TDRW and TRL, the correlation coefficient in the middle and late growth periods was relatively large, while for TRSA and TRV, there was little difference during the growth period except at the heading stage. All the correlation coefficients reached a maximum at the heading stage. In addition, in terms of their correlations with grain yield, the root parameters at the early growth stage ranked as TRV > TRSA > TRL > TDRW, while at the late growth stage, they ranked as TDRW > TRL > TRSA > TRV. In addition, the above mentioned four root parameters were positively and significantly correlated with soil water content, soil water use efficiency and precipitation water use efficiency except for the relationship between TDRW and soil water content (see Supplementary Table S1 online).

## Discussion

### Effects of tillage and irrigation on the root spatiotemporal distribution

SS reduces soil compaction, increases DRWD and promotes root growth^26^. This study found that the promotion effects of tillage on root structure were embodied in the following aspects: the root parameter peak in the RT treatment appeared earlier, and all the growth characteristic parameters in the 1 m soil layer reached a maximum at the heading stage, in contrast, SS delayed root senescence, and the peaks of TDRW, TRL, TRSA, and TRV were postponed to the anthesis stage. This may be due to the increased soil water consumption, precipitation water use efficiency and irrigation water use efficiency in SS treatment^6^. Applying irrigation too early or too late during early growth is not conducive to root growth in the middle and late stages, and properly prolonging the irrigation date in the early growth stage can result in high yield and highly efficient root traits^27^. Studies have shown that irrigation at the jointing and anthesis stages could increase the absorption area of deep roots^28^. However, this study showed that the W1 irrigation mode slowed root senescence in the deep soil layer, because irrigation only at jointing can help roots penetrate the soil and increase the absorption and utilization of deep water^29^. Excessive irrigation causes much ineffective evapotranspiration at the late growth stage, and thus water use efficiency greatly decreases^6^. Furthermore, an appropriate irrigation deficit can enhance the soil enzyme activity in deep soil^30^.

The influence of tillage under different soil layers on root configuration remain unclear. We found that, with increasing soil depth, the maximum appearance time of each root parameter was delayed overall. The parameter peak in the deep soil layer under RT was postponed to the anthesis stage, while that under SS was postponed to the filling stage, and the post-peak attenuation decrease was smaller than that in the RT treatment. This may because deep ploughing can reduce the soil bulk density of the 20–40 cm soil layer in lime concretion black soil and increase the soil porosity and field water-holding capacity of the 20–40 cm layer^31^. And root growth parameters are determined by soil bulk density^32^. These suggest that tillage methods can promote root growth by improving soil structure. Previous studies usually focused on the effects of single factor (tillage or irrigation) on root growth of winter wheat^33–36^. However, there are few literatures about double factors and their interactions on root structures in different soil layers of winter wheat in lime concretion black soil. Ali and his colleagues found that, under the condition of 200 mm rainfall in a dry-land farming system, the ridge furrow rainfall harvesting technique with 150 mm deficit irrigation could significantly promote the physiological morphology of root system in the top 40 cm soil layer, resulting in higher grain yield^37^. In another experiment, no-tillage promoted the accumulated density in shallow root system under drought condition and this is highly dependent on surface irrigation water^38^. In this study, however, the regulation effects of tillage and irrigation on the root system were mainly manifested in the root system below 60 cm soil layer. The spatiotemporal distribution of root growth parameters in the 1 m soil layer under SSW1 was the best. Under this treatment, the peak appearance times of RLD, RSA and RV in deep soil layers were delayed. This study also indicated that under SSW1, the growth peak of the deep root system occurred later, and the post-peak attenuation decline was slowed. Thus, we consider a good root spatiotemporal distribution to be the main morphological or physiological factor causing high yield.

### Effects of tillage and irrigation on grain yields

Xu et al.^39^ demonstrated that irrigation at anthesis and jointing with 150 mm of water was the best irrigation mode in the North China Plain. However, Meng et al.^40^ considered that irrigation applied at the wintering and jointing stages is good for nitrogen accumulation and utilization. If irrigation at anthesis was applied based on the pattern mentioned above, the proportion of accumulated nitrogen transferred to the grain would be significantly reduced, which would lead to decreases in nitrogen fertilizer utilization and yield. Similar results in single factor have previously been reported by Meng and his colleagues^40^, indicating that the yield in the treatment of irrigation at jointing stage was the highest. However, two tillage treatments on the basis of single irrigation factor have been added in our study. Compared with RT, SS increases the transpiration rate, net photosynthetic rate and water potential of flag leaves^41^. The results of our two-factor treatment showed that the yield in SSW1 treatment was highest in the lime concretion black soil of southeastern Henan. Zhang et al.^42^ reported that the TRSA, TRV and total number of root tips were significantly positively correlated with yield. In our study, root growth parameters were positively correlated with grain yields at different degrees, because there are positive correlations among root growth parameters, water use efficiency and nitrogen use efficiency^43^. In addition, root parameters in our study were significantly and positively correlated with soil water content, soil water use efficiency and precipitation water use efficiency. Thus, it has been speculated that the root growth parameters could significantly affect the water and nitrogen absorption of winter wheat, then regulate the kernels per spike and the thousand-grain weight, and ultimately affect grain yield. In particular, SSW1 can effectively delay root system senescence, maintain high root biomass and root length, and increase RSA and RV in the late growth stage, which play an important role in achieving high yield.

### Root contributions at different soil layers to grain yields

Guo et al.^44^ showed that the roots in soil layers above 20 cm and below 1 m contributed mostly to yield, especially the wheat plant roots below 1 m, which were more important for yield when wheat suffered drought at the late stage of growth. Moreover, Wang et al.^45^ showed that the root system of summer maize in the 0–40 cm soil layer had the greatest influence on nitrogen accumulation and transport after anthesis and thus had the greatest impact on yield. However, the root system in the soil layer below 80 cm had a significant influence on the thousand-grain weight of summer maize. This study revealed that the root system at 0–40 cm soil depth made the largest contribution to yield, but the roots at the lower and middle depths below 40 cm contributed as much as 21.68% to yield. Yield mainly depends on the shallow root system with abundant root biomass, but the deep root system has significant effects on the yield of crops such as wheat and rice^46,47^. Therefore, the roots in deep layers play an important role in determining yield. For this reason, in production practices, promoting deep rooting and maintaining the quantity and activity of the deep root system should be key to achieving high and stable wheat yields.

## Conclusions

In the lime concretion black soil, the root structures and distribution characteristics of winter wheat were positively related to soil water content, root water absorption characteristics and grain yields. SSW1 should be used as a high-yield and high-efficiency cultivation mode for winter wheat, and it was characterized with moderate root growth and distribution at the surface and upper soil layers but relatively large root amounts at the middle and lower soil layers. With this cultivation model, the growth peak of deep roots appeared later, and the post-peak attenuation decline was postponed. With this root distribution characteristic, water storage consumption at the middle and lower soil layers was effectively increased, much root biomass at late growth stages was sustained, water requirement during grain filling period was achieved, and water use efficiency was increased, resulting in the promoted dry matter productions and grain yields. In conclusion, the root proportions at deep soil layers can play partial roles in the potential of wheat grain yields. Improving the root distribution and absorption capacity at deep soil layers during cultivation helps obtain high grain yields and high water use efficiency.

## Materials and methods

### Study site

A field experiment was conducted in lime concretion black soil for two successive years from 2015 to 2017 at the 14th sub-farm of Shangshui County, Henan Province, China (33° 32′ N, 114° 29′ E). This experimental site is in a warm temperate continental monsoon climate, and the soil type is characterized with high viscosity, dry shrinkage and wet expansion, and shallow groundwater, as described in one previously published literature^6^. In our study site, there exists the lime concretion at 60–80 cm soil layers in our study site, and deep soil layers are relatively soft. The wilting coefficient and the available water content of the soil were 8.02% and 23.42%, respectively. Moreover, the annual average temperature in this area was 14.5 ℃, the accumulated sunshine duration was 2072.3 h, the average frost-free season was 223 d, and the annual average precipitation was 784.1 mm (meteorological data for 1997–2017, provided by the Shangshui Meteorological Bureau). The nutrient content of the plough layer soil was measured in 2015 before wheat sowing. The values observed in the 0–20 cm soil layer were as follows: organic matter, 21.32 g·kg^−1^; total nitrogen, 1.36 g·kg^−1^; Olsen phosphorus, 16.83 mg·kg^−1^; available potassium, 214.61 mg·kg^−1^; and pH, 7.23. Those in the 20–40 cm soil layer were as follows: organic matter, 16.06 g·kg^−1^; total nitrogen, 1.23 g·kg^−1^; Olsen phosphorus, 8.58 mg·kg^−1^; and available potassium, 163.26 mg·kg^−1^. The soil bulk density data before and after tillage are shown in Supplementary Table S2 online.

The precipitation amounts during the wheat growing periods in 2015–2016 and 2016–2017 were 281.3 mm and 360.9 mm, respectively. Precipitation and irrigation amounts at different growth stages in the two years are shown in Supplementary Table S3 online. The field water-holding capacity is shown in Supplementary Table S4 online. Solar radiation and daily average air temperature during the wheat growing stages were 12.80 MJ·m^−2^·d^−1^ and 9.52 ℃ (2015–2016), and 13.51 MJ·m^−2^·d^−1^ and 10.81 ℃ (2016–2017), respectively.

## Experimental design

### Field experiment

The crop previously grown in the experimental field was corn, and the straw was crushed and returned to the field. Wheat cultivar Zhoumai 27 used in this experiment is a commercial cultivar, which has been authorized by the China government in 2011 to be permitted to plant in China. In both years, wheat sowing was delayed by continuous rain before sowing. The tillage and sowing dates of the two years were October 27 and October 30 (2015–2016) and November 1 and November 4 (2016–2017), respectively. The sowing rate in both years was 217.50 kg·ha^−1^. The conditions at field emergence under different treatments and soil moisture content before sowing in the two-year test are shown in Supplementary Table S5 online. A Nonghaha 2BXF-12 wheat seeder was used for seeding, with a row spacing of 20 cm. A split block experiment was conducted. The main treatment was tillage, including SS and RT. The depths of SS and RT were 30–33 cm and 15–17 cm, respectively. The secondary treatment was irrigation, with three levels: W0, W1 and W2. Six treatments in different tillage and irrigation were placed randomly after distinguishing the main and secondary plots. Each irrigation amount was controlled at 75 mm, which was measured by a water meter. Impermeable planting areas with intervals of two metres were established between secondary treatments. Pure N (240 kg·ha^−1^), P_2_O_5_ (120 kg·ha^−1^), and K_2_O (90 kg·ha^−1^) were applied throughout the whole growth period of wheat. The phosphate and potash fertilizers were applied basally before sowing. Similar to W1 and W2 treatments, half of the N fertilizer amounts in W0 treatment were applied before sowing, and the remaining fertilizers were applied at jointing. Because precipitation occurred at jointing stage in the two wheat growth seasons, no irrigation was been performed in W0 treatment, and the remaining N fertilizers were applied during the jointing period according to the precipitation or the soil moisture. Each treatment was repeated three times, and the area of each plot was 60 m^2^ (10 m × 6 m). Pest, weed and disease control was carried out according to local guidelines.

### Soil column experiment

In-situ soil column experiment was set in the field experiment. Before soil preparation, one repeated planting area of the field experiment was selected to plot a certain area. Soil in 1.5 m deep was turned over to the ground at six levels: 0–20 cm, 20–40 cm, 40–60 cm, 60–80 cm, 80–100 cm, and below 100 cm. Soil above 1 m was mixed thoroughly by layers, and soil bulk density of each soil layer was measured. The soil column was made according to the method of Fan et al.^48^, and appropriate improvements were made. Specific device was shown in Supplementary Fig. S2 online. In order to facilitate root extraction, root breaking and washing during wheat growth, PVP plastic tube with a height of 210 cm and an inner diameter of 40 cm was cut in half along the axial direction. Then cut into 150 cm, 80 cm, and 40 cm according to the root cutting height. The round tube was fixed with a round steel clip after cutting and marked at the pre-cut root. The gap was sealed with glass glue to make it impermeable. The tube bottom was fastened with a leaky iron plate, and a wire rope was fixed on it to facilitate lifting the soil column from the soil pit. Put the prepared soil column into the soil pit vertically. In order to reduce differences in soil layers, the field soils at each layer were separately sampled and transferred to the related layers of the PVP plastic tube used for the soil column experiment. Actual soil moisture contents were measured before loading soil layers. Soil of 0–20 cm should be mixed thoroughly according to the base fertilizer amount for each treatment in the field test and then backfilled. Root-breaking treatments were performed in the 0 cm (CK), 40 cm (T-40), and 80 cm (T-80) soil layers at anthesis. Three soil columns were made for each treatment, and a total of 54 soil columns were made by combining six treatments of field experiment. Soil around soil column was dug out at anthesis and all the roots were cut off at the mark with electric wire saws. In order to facilitate root cutting, soil columns with the same treatment were arranged together. Seedling amounts (30) in each soil column were same as the field experiment, and the sowing, topdressing and irrigation time were the same as the field. The wheat variety used was Zhoumai 27.

## Contents of determinations and methods

### Root parameters

Root samples were collected from the 0–100 cm soil layer in each plot at the jointing, heading, anthesis, filling and maturity stages, and every 20 cm of depth was regarded as one soil layer. The sampling method was the same as that used by Wang et al.^7^. After processing, the root parameters such as root length, root surface area and root volume in different soil layers were obtained by root system analysis software (WinRHIZO 2008). The root was dried with absorbent paper and then placed in an oven at a constant temperature of 80 ℃ to obtain the root dry weight. The dry root weight density (DRWD, g·m^−3^), root length density (RLD, cm·cm^−3^), root surface area per unit area (RSA, m^2^·ha^−1^) and root volume per unit area (RV, m^3^·ha^−1^) in different soil layers were determined by Eqs. (1), (2), (3) and (4), respectively^49–52^:

1 $$DRWD=\frac{M}{V}\times {10}^{6}$$

2 $$RLD=L/V$$

3 $$RSA=A/S\times {10}^{8}$$

4 $$RV=V{^{\prime}}/S\times {10}^{8}$$

Total dry root weight (TDRW, g·m^−2^), total root length (TRL, cm·cm^−2^), total root surface area (TRSA, m^2^·ha^−1^) and total root volume (TRV, m^3^·ha^−1^) refer to the sums of root weight, root length, RSA, and RV per unit soil area in different soil layers^53^. They were determined by Eqs. (5), (6), (7) and (8), respectively:

5 $$TDRW={\sum }_{i=1}^{n}{M}_{i}/S\times {10}^{4}$$

6 $$TRL=\sum_{i=1}^{n}{L}_{i}/S$$

7 $$TRSA=\sum_{i=1}^{n}{A}_{i}/S\times {10}^{8}$$

8 $$ TRV = \sum\nolimits_{{i = 1}}^{n} {V_{i}^{\prime } } /S \times 10^{8} $$

where M, L, A, V', V and S are root dry weight (g), root length (cm), root surface area (m^2^), root volume (cm^−3^), soil volume (cm^−3^) and soil area (cm^2^), respectively. The soil layer number is represented by i. There are five layers in total, and n is the total number of soil layers. Moreover, M_i_, L_i_, A_i_ and V_i_' are the root dry weight, root length, root surface area and root volume of the i-th layer, respectively.

### Grain yields and components

In each plot, the 6 m^2^ (2 m × 3 m)-wheat plants were manually harvested to measure grain yields. The fixed sampling points for one-metre double rows of each treatment were investigated at maturity to obtain the spike number. Fifty representative stems were selected for each repeated treatment in each plot during the harvest period, packed into mesh bags, marked and then taken back to the laboratory to measure the kernel number per spike. Grain weight was measured after threshing and drying and converted into actual yield according to a 13% water content.

### Yield contribution rate

Wheat samples from the soil column experiment were harvested at maturity to measure wheat yield and three factors of yield. No root cutting (CK), cutting of wheat roots at 40 cm below the surface (T-40) and cutting of roots at 80 cm below the surface (T-80) are represented by Y_0_, Y_40_ and Y_80_, respectively. C_40_, C_40-80_ and C_80_ are the yield contribution rates of roots in the 0–40 cm, 40–80 cm, and below 80 cm soil layers, respectively. They were determined by Eqs. (9), (10) and (11), respectively:

9 $${C}_{40}={Y}_{40}/{Y}_{0}\times 100$$

10 $${C}_{40-80}=({Y}_{80}-{Y}_{40})/{Y}_{0}\times 100$$

11 $${C}_{80}={(Y}_{0}-{Y}_{80})/{Y}_{0}\times 100$$

### Statistical analysis

Excel 2013 and Origin 2018 were used for data processing and graphing. Statistical analyses, significance tests (Duncan's test) and correlation analysis (Pearson) were performed using SPSS 23.0.

## Supplementary Information

Supplementary Information
