# Supplementary material for: Tillage and irrigation increase wheat root systems at deep soil layer and grain yields in lime concretion black soil
Source: Sci Rep. 2021 Mar 18;11:6394. doi: 10.1038/s41598-021-85588-6 (PMC7973502; doi:10.1038/s41598-021-85588-6)
Supplement: Supplementary file 1 — Supplementary Information [file 41598_2021_85588_MOESM1_ESM.pdf]

Tillage and irrigation increase wheat root systems at deep soil layer and grain yields in lime concretion black soil

Jinfeng Wang[1,2], Zhuangzhuang Wang[1,2], Fengxu Gu[1,2], Huan Liu[1,2], Guozhang Kang[1,2,3], Wei Feng[1,2,3], Yonghua Wang[1,2,3,*], Tiancai Guo[1,2,3,*]

Supplementary Information

Supplementary Table S1. Correlation analysis among root parameters and soil water content, soil water use efficiency and precipitation water use efficiency under different tillage practices and irrigation regimes.

Supplementary Table S2. Soil bulk density of different soil layers before and after tillage.

Supplementary Table S3. Precipitation and irrigation amounts at different growth stages in two wheat growing seasons.

Supplementary Table S4. Field water-holding capacity of different soil layers in the experimental field in the two years.

Supplementary Table S5. Basic seedling number under different treatments and soil moisture content at different soil depths before sowing in the two years.

Supplementary Figure S1. Correlation analysis of the root growth parameters of winter wheat under different tillage practices and irrigation regimes.

Supplementary Figure S2. Schematic diagram of soil column structure.

Supplementary Table S1. Correlation analysis among root parameters and soil water content, soil water use efficiency and precipitation water use efficiency under different tillage practices and irrigation regimes.

Item	Soil water content	Soil water use efficiency	Precipitation water use efficiency
Total dry root weight	0.31	0.37*	0.71**
Total root length	0.56**	0.52**	0.37*
Total root surface area	0.52**	0.43**	0.33*
Total root volume	0.52**	0.40*	0.38*

Note: * represents significant correlation (P<0.05), ** represents extremely significant correlation (P<0.01).

Supplementary Table S2. Soil bulk density of different soil layers before and after tillage.

Year	Treatment	Time measured	Soil bulk density (g cm^{-3})				
			0-20 cm	20-40 cm	40-60 cm	60-80 cm	80-100 cm
2015-2016	SS	Before tillage	1.38c	1.44c	1.42b	1.55a	1.67a
		After harvest	1.37c	1.47b	1.39c	1.45c	1.57c
	RT	Before tillage	1.43b	1.53a	1.45a	1.55a	1.67a
		After harvest	1.48a	1.46bc	1.25d	1.51b	1.64b
2016-2017	SS	Before tillage	1.36c	1.46d	1.40b	1.44c	1.58c
		After harvest	1.44b	1.50b	1.41b	1.52b	1.62b
	RT	Before tillage	1.50a	1.48c	1.35c	1.51b	1.63b
		After harvest	1.49a	1.52a	1.43a	1.57a	1.65a

Note: data followed by different lowercase letters within any column indicate that the difference is significant at the P = 0.05 level. The same as below.

Supplementary Table S3. Precipitation and irrigation amounts at different growth stages in two wheat growing seasons.

Year	Treatment		Precipitation amount				Irrigation amount	
			Sowing-Wintering (mm)	Wintering-Jointing (mm)	Jointing-Anthesis (mm)	Anthesis-Maturity (mm)	Jointing (mm)	Anthesis (mm)
2015-2016	SS	W0	99.34	37.31	43.41	101.30	0	0
		W1	99.34	37.31	43.41	101.30	75	0
		W2	99.34	37.31	43.41	101.30	75	75
	RT	W0	99.34	37.31	43.41	101.30	0	0
		W1	99.34	37.31	43.41	101.30	75	0
		W2	99.34	37.31	43.41	101.30	75	75
2016-2017	SS	W0	136.65	63.55	63.13	97.64	0	0
		W1	136.65	63.55	63.13	97.64	75	0
		W2	136.65	63.55	63.13	97.64	75	75
	RT	W0	136.65	63.55	63.13	97.64	0	0
		W1	136.65	63.55	63.13	97.64	75	0
		W2	136.65	63.55	63.13	97.64	75	75

Supplementary Table S4. Field water-holding capacity of different soil layers in the experimental field in the two years.

Year	Treatment	Field water-holding capacity (%)				
		0-20 cm	20-40 cm	40-60 cm	60-80 cm	80-100 cm
2015-2016	SS	34.26	33.35	32.29	31.36	28.27
	RT	34.02	33.17	31.86	29.66	26.91
2016-2017	SS	34.19	34.03	32.26	29.71	27.57
	RT	33.50	33.43	31.39	29.23	28.42

Supplementary Table S5. Basic seedling number under different treatments and soil moisture content at different soil depths before sowing in the two years.

Year	Treatment		Basic seedling number (10^4 ha^{-1})	Soil moisture content (%)				
				0-20 cm	20-40 cm	40-60 cm	60-80 cm	80-100 cm
2015-2016	SS	W0	283.10c	31.89a	32.73a	30.64a	23.96a	20.50a
		W1	285.37c	31.89a	32.73a	30.64a	23.96a	20.50a
		W2	308.11b	31.89a	32.73a	30.64a	23.96a	20.50a
	RT	W0	251.04b	32.87b	33.66b	31.80b	25.38b	22.32b
		W1	258.84b	32.87b	33.66b	31.80b	25.38b	22.32b
		W2	278.11a	32.87b	33.66b	31.80b	25.38b	22.32b
2016-2017	SS	W0	333.12b	30.42b	31.22b	29.22b	22.86ab	19.55b
		W1	339.37ab	31.91b	32.75b	30.66b	23.98bc	20.51b
		W2	344.30ab	31.91a	32.75a	30.66a	23.98a	20.51a
	RT	W0	331.42b	31.85c	32.62c	30.81c	24.60d	21.63d
		W1	337.10ab	31.77b	32.54b	30.74b	24.53c	21.57c
		W2	338.81a	32.55b	33.34b	31.50b	25.14c	22.10c

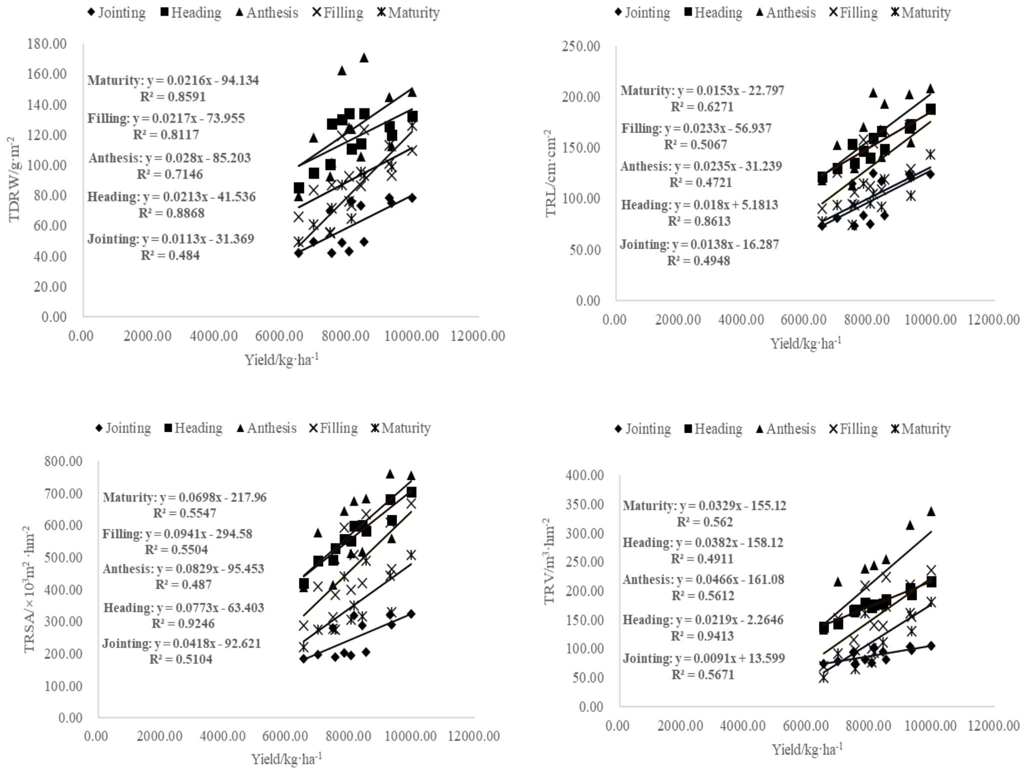

Supplementary Figure S1. Correlation analysis of the root growth parameters of winter wheat under different tillage practices and irrigation regimes.

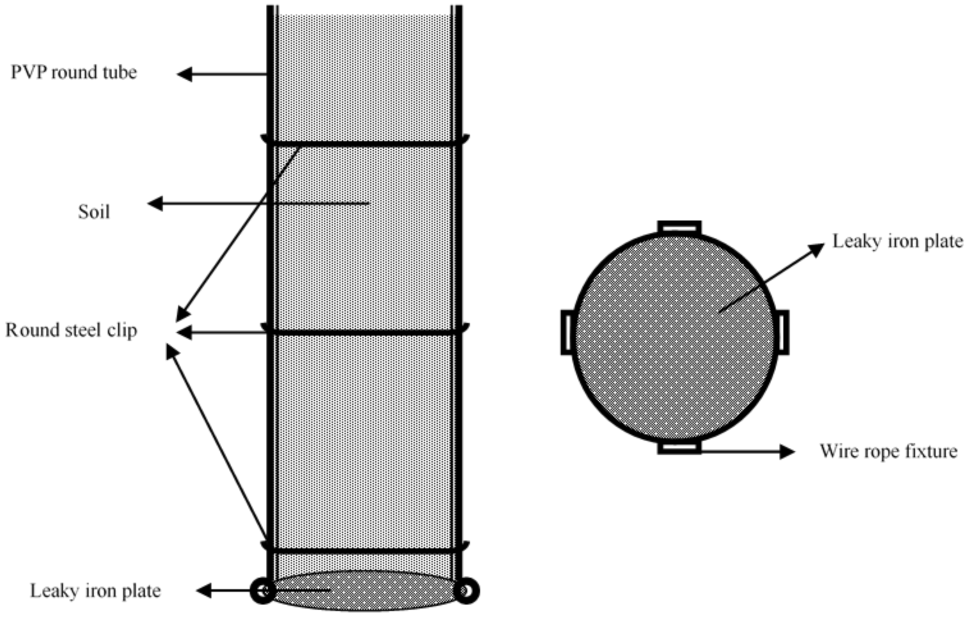

Supplementary Figure S2. Schematic diagram of soil column structure
